# Enhancing Resource Sharing and Access Control for VNF Instantiation with Blockchain

**DOI:** 10.3390/s23239343

**Published:** 2023-11-23

**Authors:** Anwei Dong, Xingwei Wang, Bo Yi, Qiang He, Min Huang

**Affiliations:** 1 College of Computer Science and Engineering, Northeastern University, Shenyang 110169, China; 1610550@stu.neu.edu.cn (A.D.); yibo@cse.neu.edu.cn (B.Y.); 2 College of Medicine and Biological Information Engineering, Northeastern University, Shenyang 110016, China; heqiang@bmie.neu.edu.cn; 3 College of Information Science and Engineering, Northeastern University, Shenyang 110819, China; mhuang@mail.neu.edu.cn

**Keywords:** NFV, VNF, resource sharing, game model

## Abstract

In the realm of Network Function Virtualization (NFV), Virtual Network Functions (VNFs) are crucial software entities that require execution on virtualized hardware infrastructure. Deploying a Service Function Chain (SFC) requires multiple steps for instantiating VNFs to analyze, request, deploy, and monitor resources. It is well recognized that the sharing of infrastructure resources among different VNFs will enhance resource utilization. However, conventional mechanisms for VNF sharing often neglect the interests of both VNF instances and infrastructure providers. In this context, this paper presents a blockchain-based framework that focuses on resource sharing and access control, with a particular emphasis on ensuring profitability during VNF instantiation. Additionally, a resource sharing game model and a novel greedy matching algorithm are introduced to optimize the benefits for both VNF instances and infrastructure resource providers. Furthermore, a blockchain-based access control mechanism is designed to securely store keys and provide fine-grained access control. The experimental results demonstrate that the proposed resource sharing game model and greedy matching algorithm promote healthy competition among resource owners and facilitate effective bargaining between resource owners and infrastructure providers. In comparison to the standard Stackelberg game solution, our proposed method achieves up to an 8.1 times performance improvement while sacrificing fewer optimal social utility values. Furthermore, compared to other CP-ABE methods, the proposed approach enhances security within a blockchain-based framework while maintaining an excellent encryption efficiency and a moderate decryption efficiency.

## 1. Introduction

Network Function Virtualization (NFV) offers a flexible and scalable approach for the deployment and management of network functions. In the traditional paradigm, network functions were reliant on dedicated hardware devices. However, NFV decouples these functions from specialized hardware, enabling them to operate as software on general-purpose servers [1]. Consequently, various network functions within an IP network can now be configured and managed with increased flexibility and efficiency.

Through the adoption of NFV, network operators and providers gain the ability to easily adjust, upgrade, and introduce new services without being tied to specific hardware dependencies. This adaptability is pivotal for meeting evolving network demands and accommodating increasing traffic. Consequently, integrating NFV with IP networks represents an innovative approach for constructing a more intelligent, flexible, and scalable network infrastructure.

VNFs necessitate execution on virtualized hardware infrastructure, such as virtual machines, containers, or other virtualization platforms, as discussed in a comprehensive review by Kaur et al. [2]. When implementing a Service Function Chain (SFC) within this context, VNFs must go through distinct steps during instantiation.

Requirements Analysis and Planning: This initial phase involves identifying essential VNFs and determining the computing, storage, networking, and other resource requirements for each VNF.

Resource Request: The request for resources, including CPU, memory, and bandwidth, that are necessary for VNFs, must be submitted to the infrastructure or cloud service provider.

Resource Allocation: Resource allocation is carried out based on the submitted resource requests and the availability of resources.

VNF Deployment: After resource allocation, the VNFs are deployed into their designated virtualized instances, such as virtual machines or containers.

Configuration and Optimization: Each VNF undergoes configuration and optimization to ensure the efficient utilization of allocated resources and optimal performance.

Monitoring and Management: Regular and systematic monitoring of VNF performance, resource utilization, and security is a non-negotiable imperative. This enables timely adjustments and optimizations, contributing to the robustness of the entire NFV ecosystem.

In conventional NFV deployments, individual VNFs typically monopolize underlying resources to preempt conflicts. In such instances, service providers deploying service function chains can bypass concerns about interactions among different VNFs, simplifying deployment into a straightforward rental model. However, with the expanding array of network functions, particularly in the context of the burgeoning 5G and evolving 6G networks, the deployment of intricate service function chains has garnered attention. This proliferation has given rise to diverse VNF types, creating a dynamic landscape. Due to fluctuating user demands for various network services at different times, service providers often allocate excess basic resources to ensure a seamless user experience during peak traffic periods. Unfortunately, this practice often leads to the wastage of resources and higher costs. Conversely, infrastructure providers grapple with limitations in managing complex network traffic and providing flexible resource configurations.

Current research in resource sharing [3,4,5,6,7,8,9,10,11,12,13] predominantly concentrates on two key dimensions. The first involves the judicious placement of VNFs, which requires determining the optimal locations and quantities within the network to maintain service quality and improve resource utilization. This encompasses an examination of infrastructure resource sharing among multiple VNFs co-located on the same node, with the overarching goal of achieving efficient infrastructure resource utilization. The second dimension centers on refining service chaining methodologies. This involves carefully scheduling data packets from various business chains on a shared VNF, enabling efficient resource sharing and promoting VNF sharing. Nevertheless, these methodologies often neglect the inherent interests among different VNF entities and infrastructure providers. As the adage goes, “all’s fair in love and war.” Devoid of appropriate incentives, even the most adept resource-sharing strategies pose implementation challenges. Hence, it becomes imperative to incorporate economic incentives into the VNF instantiation phase to maximize the benefits for both infrastructure providers and VNF entities involved in resource sharing.

Access control is a critical component in NFV, which function as a pivotal mechanism for safeguarding sensitive data and resources. This mechanism involves various operations that include different subjects, such as users, roles, services, etc. Its significance lies in its ability to regulate access to distinct objects, including files, devices, and services, while concurrently ensuring the integrity, confidentiality, and availability of resources [14]. In multi-tenant environments that share common infrastructure and resources, access control policies can be vulnerable to inconsistencies and conflicts. This can amplify concerns regarding resource isolation and protection. Within such contexts, the presence of malicious or unauthorized entities poses a looming threat, with the potential to compromise sensitive data, disrupt normal service operations, and instigate severe consequences. These consequences can range from data breaches to service interruptions and performance degradation.

**Figure 1:**
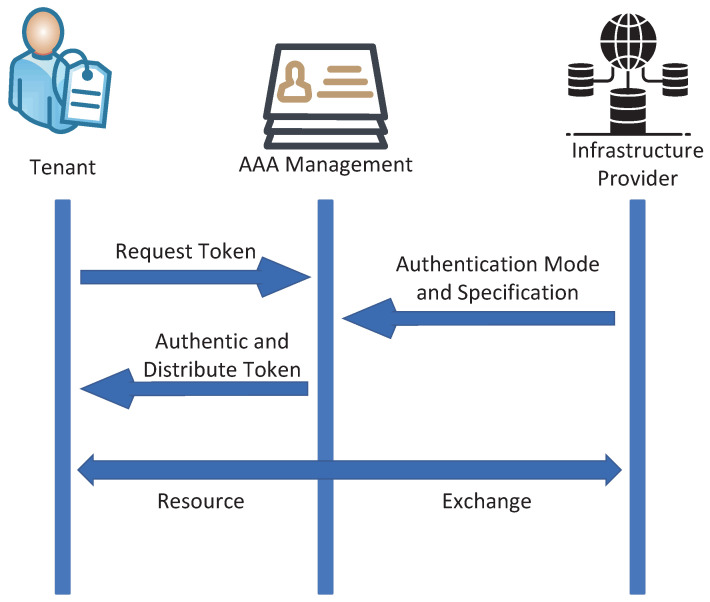
Traditional access control model.

Prior investigations in the field of NFV resource sharing have notably neglected the examination of robust access control strategies, despite their pivotal significance. Other research on access control [15,16,17,18] primarily focuses on Role-Based Access Control (RBAC), Attribute-Based Access Control (ABAC), and Policy-Based Access Control (PBAC). To ensure precise subject identification and verification within the NFV system, trusted identity management and authentication mechanisms are commonly deployed. These mechanisms utilize various tools, such as digital certificates, tokens, passwords, and similar techniques, as illustrated in Figure 1. Nevertheless, in many conventional approaches, it is common to rely on third-party involvement for key distribution. If the third party lacks trustworthiness, data security is severely compromised.

Blockchain is a decentralized and distributed ledger technology that enables secure and transparent record-keeping of transactions across a network. Each transaction, or “block”, is linked to the previous one through a cryptographic hash, which forms a chain of blocks. This immutability and consensus mechanism makes it extremely resistant to tampering or unauthorized alterations. Blockchain is most commonly associated with cryptocurrencies such as Bitcoin, where it serves as the underlying technology for a secure and decentralized financial system. However, its applications extend beyond finance to various industries, including supply chain management, healthcare, and smart contracts, offering enhanced transparency, traceability, and trust in digital transactions [19,20,21,22,23,24]. Notably, recent studies have explored the utilization of blockchain technology in fields associated with the placement, addressing, and resource allocation of VNFs [25,26,27,28].

Game theory, a mathematical discipline [29], delves into the strategic interactions among decision makers, commonly referred to as players, across diverse scenarios. This branch involves analyzing the choices made by players, known as strategies, and gaining a comprehensive understanding of the resulting outcomes and impacts on each participant. Whether in the realm of economic competition, political decision making, or biological interactions, game theory offers a versatile framework for examining situations wherein individuals or entities navigate decisions within dynamic and interactive environments. Its applications traverse disciplinary boundaries, providing valuable insights into cooperation, competition, and the intricate interplay of decisions within human and natural systems. Notably, in recent years, game theory has been frequently applied to delineate VNF chains and formulate strategies for the allocation of VNF resources [30,31,32,33,34,35].

In light of the previously discussed issues and challenges, this paper proposes a novel mechanism based on blockchain technology. This mechanism is specifically designed for resource sharing and access control during the VNF instantiation stage. The primary contributions of this research are as follows:

First, we present a comprehensive system framework for VNF resource sharing and access control based on blockchain. This framework delineates essential processes and methodologies for resource request and deployment during the VNF instantiation process.

Second, we delve into the intricate dynamics between VNF instances and infrastructure resource providers, which leads to the formulation of a resource-sharing game model. The primary goal is to optimize the benefits for both infrastructure resource providers and VNF instances involved in resource sharing. To operationalize this model, we introduce a greedy matching algorithm.

Third, we design and implement a blockchain-driven VNF attribute-encrypted access control mechanism. This mechanism leverages blockchain technology and attribute-based encryption, incorporating ciphertext policy enforcement. Furthermore, we integrate the use of a Bloom Filter to obscure the access policies of the instantiated VNF.

Ultimately, we conduct comprehensive simulation experiments using the Open Network Automation Platform (ONAP) and Ethereum to rigorously evaluate the proposed mechanism and its associated algorithms. The simulation results unequivocally confirm the effectiveness of the proposed mechanism, surpassing the performance benchmarks set by existing methodologies.

The subsequent sections of this paper are structured as follows: Section 2 provides a review of relevant prior research. Section 3 expounds upon the framework for the blockchain-based resource sharing and access control system. The formulation of the resource sharing game model and the accompanying greedy matching algorithm are presented in Section 4 and Section 5, respectively. Section 6 offers insights into the access control algorithm based on blockchain and attribute encryption. Section 7 is dedicated to the evaluation of performance, while the conclusion of this paper is concluded in Section 8.

## 2. Related Work

According to the findings of the literature survey, the strategic placement of VNFs encompasses a multifaceted approach. This entails a thorough analysis of the necessary resources, the formulation of strategies for resource allocation, the resolution of conflicts pertaining to resource sharing, the optimization of performance, the balancing of workloads, the dynamic adjustment of resources, and the implementation of secure isolation mechanisms. These efforts are all directed towards achieving the twin objectives of maximizing resource utilization and fulfilling network functionality requirements. Huang et al. [3] introduced AutoVNF, an automated mechanism for optimizing VNF deployment. This mechanism incorporated a resource-sharing mode and an automated resource allocation mechanism, which effectively support multiple VNFs sharing resources on a single node and dynamically allocating available nodes to VNF requests. Cohen et al. [4] proposed two approximate algorithms to address the VNF placement problem. One for cases without capacity constraints and another for cases with capacity constraints. The primary objectives of these algorithms were to minimize the distance between users and service nodes and to reduce the deployment cost of VNFs. Sun et al. [5] presented a method for optimizing the placement of VNFs. The method took into account the resource sharing among VNFs and the stochastic characteristics of Poisson arrival traffic. This approach utilized a queuing model to examine queueing delays within the VNF queue, thereby formulating the VNF placement problem as a 0–1 quadratic fractional programming challenge. This method addressed the complexities of balancing service quality and placement expenses across diverse traffic categories in resource-sharing VNFs. Savi et al. [6] introduced a method that leverages Integer Linear Programming (ILP) and Heuristic Computing Algorithm (HCA) for optimizing VNF placement and SFC embedding. This approach considered the performance loss due to the sharing of processing resources in a multi-core CPU architecture, which includes the associated cost increase and context-switching overhead. The main objective of this approach was to minimize the number of activated NFV nodes, thereby reducing the implementation cost associated with NFV.

With the widespread adoption of machine learning [36], numerous studies have been proposed to achieve intelligent VNF mapping. In their work, Sun et al. [7] advocated for a dynamic resource allocation scheme grounded in VNFs. This scheme leveraged online learning techniques to forecast user mobility patterns and allocate resources according to the heat generated by base stations. The authors introduced a supplementary mechanism that reallocates idle resources from demand-surplus base stations to demand-deficient ones. This mechanism prioritized the requirements of the latter group. Mu et al. [8] presented an approach based on Deep Reinforcement Learning (DRL) to optimize VNF placement. This study adopted a holistic approach to address the issue of data center server energy consumption and performance interference among VNFs. The objective is to minimize the overall server energy consumption while ensuring that the performance of each VNF exceeds a predetermined threshold. Basu et al. [9] proposed a machine learning-based methodology that integrates SDN and NFV to realize dynamic resource sharing in 5G-assisted unmanned aerial vehicle networks. The approach employed two regression models, Support Vector Regression and Kernel Ridge Regression, to predict VNF resource requirements and dynamically allocate VNFs based on the prediction results.

VNF sharing entails the optimal utilization of a singular VNF instance to handle multiple service requests that necessitate the same category of VNF. It is also applicable in cases where a specific VNF type needs to be deployed in multiple instances to meet the requirements of a particular service. Within VNF sharing, resources assigned to a VNF specific instance are concurrently utilized by multiple data packets, thereby diminishing packet waiting times in the queue. Li et al. [10] proposed a method tailored for deploying VNFs in data centers and introduced innovative techniques such as shared redundancy and multi-tenancy. This led to the development of a Joint Deployment and Backup Scheme (JDBS). The JDBS dynamically adjusted VNF deployment and backup strategies iteratively to effectively balance Basic Resource Consumption (BRC) and Shared Redundancy Consumption (DRC), ultimately achieving an optimal equilibrium between the two. Vieira et al. [11] considered the dynamic characteristics of edge environments, incorporating factors such as resource availability, uncertainty in user requests, QoS requirements, and user mobility. They employed a time window strategy to process batches of continuously arriving service requests. The algorithm also presented a two-tier resource-sharing mechanism, which facilitates the sharing of VNF instances or SFC instances among multiple services to reduce resource consumption and associated costs. Ruiz et al. [12] introduced a Genetic Algorithm-based approach to jointly addressed VNF placement, VNF chaining, and virtual topology design. The authors leveraged collaborative capabilities among Multi-access Edge Computing (MEC) nodes to enable VNF sharing. This approach utilized a novel search strategy during the chaining process, which prioritizes the identification of available VNFs in both local nodes and Central Offices. In the absence of such resources, the search extended to the physically nearest node within the topology until all network nodes have been explored. Yi et al. [13] proposed a dynamic and flexible algorithm to address VNF shared resource allocation and rate coordination between upstream and downstream VNFs. Specifically, the algorithm considered fairness factors during VNF sharing to reduce the probability of resource contention and enhance resource utilization. Additionally, by defining a backpressure indicator for each VNF to assess its pressure status, it dynamically adjusted the processing rate between the VNF and its upstream and downstream VNFs, with the aim of optimizing the utilization of idle resources.

The study by Kumar et al. [15] offered a comprehensive examination of security concerns and resolutions pertaining to VNF within the telecommunication domain. The paper systematically analyzed potential security threats and attacks targeting various components and layers within the NFV architecture. The proposed security measures for VNFs encompass aspects such as security hardening, role-based access control, software integrity, and protection against malicious code. Gui et al. [16] presented a distinct identity and access control scheme tailored for microservices in 5G platforms, which relys on OpenID Connect and JSON Web Tokens. This scheme facilitated the authentication and authorization processes for both users and microservices, thereby enhancing the overall lifecycle management of virtualized services. A notable feature of this study resided in its practical application and comprehensive evaluation carried out within the context of the SONATA service platform. Simultaneously, Smine et al. [17] proposed an innovative approach for the correct and optimal deployment of access control policies in NFV services. The approach considered a robust insider adversary model capable of compromising one or multiple VNFs within the Management and Orchestration (MANO) framework. Furthermore, Murillo et al. [18] introduced a specialized access control framework for virtualized Industrial Control Systems (ICS). The framework incorporated an advanced policy language to clearly define the components, roles, and authorized operations within the ICS. Additionally, the system included a policy engine that facilitated the translation of high-level policies into low-level rules, enabling their execution across various virtualization platforms. The primary objective of this framework was to furnish ICS administrators with a user-friendly tool for flexibly defining and managing access control policies in virtualized ICS.

In light of the preceding analysis, contemporary research initiatives in the field of VNF resource sharing primarily focused on traffic attributes and the succession of service supply chains. Unfortunately, these efforts often neglect the crucial issue of guaranteeing a fair and just allocation of benefits among the diverse entities engaged in resource sharing. Regarding resource access control, the pertinent literature predominantly centered on enhancing extant models based on third-party authentication.

Moreover, there has been some related works on the application of blockchain in the placement and resource allocation of VNFs. Liu et al. [25] presented a blockchain-based approach that incorporates vector commitments and Succinct Non-Interactive Knowledge Proof (SNARK) techniques for VNF management. Their proposed method efficiently managed VNF dictionaries and validates queries. Taskou et al. [26] proposed a blockchain-based strategy for NFV resource allocation. Through the use of smart contracts, their approach achieved decentralized, secure, and reliable resource allocation. The paper defined two optimization problems: the NFV resource allocation problem, which aims to minimize energy consumption and resource costs for data centers, and the mining task offloading problem, which seeks to minimize energy consumption for mining users. Papadakis et al. [27] introduced a blockchain-based network service marketplace and resource orchestration mechanism to enable cross-service communication within the edge cloud. Leveraging the smart contract functionality of the Hyperledger Fabric platform, the paper automated network service interactions and lifecycle management among different tenants. Additionally, it introduced an innovative service orchestrator that utilizes the capabilities of Open Source MANO (OSM), establishing cross-service communication with minimal resource requirements and instantiation costs. Regarding the allocation and competition strategies for VNF resources, Franco et al. [28] utilized blockchain and smart contract technologies to propose a reverse auction-based solution for discovering and selecting infrastructure capable of efficiently hosting VNFs. This solution encouraged competition among Infrastructure Providers, thereby mitigating the deployment costs for VNFs while simultaneously addressing the unique needs of users. Notably, the solution leveraged the tamper-proof and auditable features of blockchain, which ensures reliable records and contract execution. An advantageous aspect of this solution was its consideration of various user and VNF requirements, such as minimum resources, geographical location, and maximum latency, rather than relying solely on pricing for infrastructure selection.

Moreover, existing literature has delved into the utilization of game theory to delineate VNF chains and formulate strategies for the allocation of VNF resources. Leivadeas et al. [30] presented an approach grounded in graph partitioning game theory to address the placement problem of VNF service chains. The method effectively implemented service chains in cloud environments. The achievement was made possible by effectively addressing server affinity, coexistence, and latency constraints. Simultaneously, the method aimed to minimize deployment costs while also achieving resource load balancing. Chen et al. [31] introduced an incentive-driven framework for VNF chains, aiming to optimize resource allocation across different layers, such as bandwidth and IT resources. This framework was specifically designed for Interconnected Data Center Elastic Optical Networks (IDC-EONs) and involved coordination among multiple agents. The framework employed a non-cooperative hierarchical game theory mechanism, where resource agents assume the role of leaders and VNF-SC users act as followers. Within the leader game, agents calculated VNF-SC service solutions for users and calculated them for configuration tasks. In the follower game, users competed for cross-layer resources based on the service solutions provided by agents, aiming to achieve a joint optimization of resource cost and service quality. Gao et al. (2022) [32] introduced a VNF placement by potential games. The objective of the method was to enhance resource allocation and improve service quality in the context of satellite edge computing. The approach modeled the VNF placement problem as a non-cooperative potential game and utilized the Nash equilibrium as the solution concept. Le et al. [33] employed a game-theoretic approach, coupled with the semi-tensor product matrix tool, to investigate the SFC routing problem. The consideration encompassed both limitations in server capacity and constraints on the minimum target rate for users. This method effectively ensured NFV server capacity constraints while meeting user rate requirements. Li et al. [34] utilized a game-theoretic approach to address the problem of embedding multiple SFCs. The methodology considered both the impact of resource sharing among different VNFs and the limitations in capacity of various NFV nodes. The objective of this approach was to minimize the end-to-end (E2E) latency for the traffic supported by each SFC while satisfying the capacity constraints of all NFV nodes. Regarding the resource allocation mechanism for VNFs, Lima et al. [35] proposed a approach to address the resource management and orchestration problem in NFV. The mechanism utilized a bilateral sealed-bid auction model, which treats users and infrastructure providers as buyers and sellers, respectively. It employed a centralized agent to match demands and bids, resulting in the optimization of the social welfare for both buyers and sellers.

Inspired by the mentioned research work, we present utility functions grounded in economic principles to systematically elucidate the intricate dynamics between infrastructure providers and participants in VNF resources. This undertaking requires the development of a cohesive game model for VNF resource sharing. Notably, our approach to access control for shared resources diverges significantly from conventional practices, as we strategically integrate blockchain technology. Although the attribute-based encryption method is utilized, the need for third-party authentication authorities is eliminated. This measure enhances the level of security and ensures the privacy protection when accessing VNF resources.

## 3. System Framework

**Figure 2:**
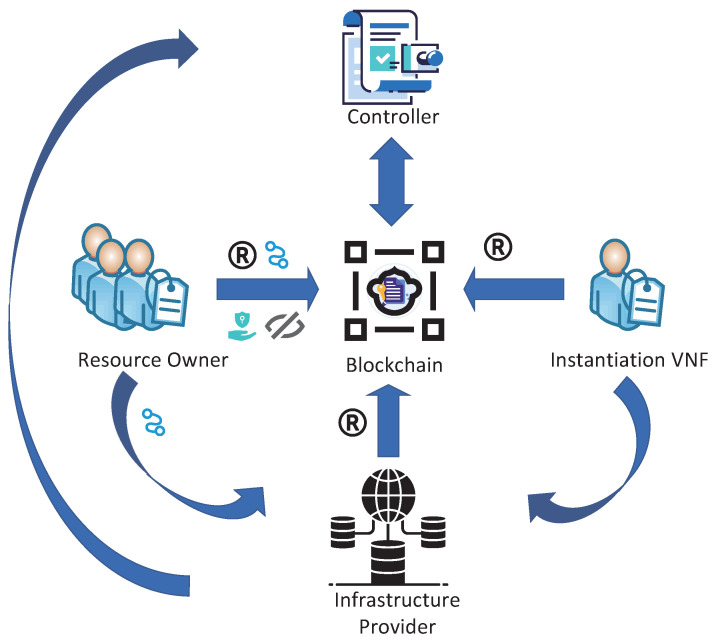
System Framework.

The proposed system, as illustrated in Figure 2, consists of five fundamental components: (i) Instantiation VNF, (ii) Infrastructure Provider, (iii) Resource Owner, (iv) Blockchain and (v) Controller.

Instantiation VNF (IV):

- The instantiation process initiates with a VNF entity requesting essential resources from the Infrastructure Provider to fulfill specific operational needs.
- Upon successful acquisition of the necessary resources, the entity is furnished with access policies meticulously tailored to its attribute set.
- These access policies play a crucial role in ensuring the enforcement of appropriate access controls within their designated time frames.

Infrastructure Provider (IP):

- Traditionally, in the context of NFV, IP has been recognized as a fundamental retailer, serving as the primary resource provider for a range of VNFs.
- IP leases resources to different VNFs based on temporal agreements.
- IP utilizes a range of strategies to effectively manage access control and bolster security measures.
- In this study, the role of IP is translated into a resource integrator. This is achieved by collecting resource utilization and preferences data from previously deployed VNFs. In this context, IP provides a hybrid resource provisioning mechanism for newly requested instantiated VNFs. This approach aims to optimize the overall system’s resource utilization and facilitate flexible resource allocation.

Resource Owner (RO):

- RO represents a currently operational VNF that is equipped with surplus resources and has a willingness to share these resources within defined temporal constraints.
- This sharing initiative is designed with the objective of generating supplementary revenue and mitigating capital expenditures.

Blockchain:

- Blockchain plays a pivotal role in the storage of cryptographic keys and the management of access control within the system.
- All entities utilize the blockchain to create and deploy smart contracts.
- These smart contracts facilitate the key distribution and access control for shared resources.

Controller:

- The controller, which is implemented through smart contracts, assumes the responsibility of monitoring and managing resources throughout the entirety of the network infrastructure.
- The RO periodically transmits pertinent information to the controller, thereby facilitating the efficient monitoring of the overall status of network resources.

Figure 2 illustrates the process of information exchange among the instantiation VNF, Infrastructure Provider (IP), and Resource Owner (RO) through the utilization of a blockchain-based platform that incorporates three contracts.

Initially, it is required for the RO, IP, and IV to complete the registration of external accounts and implement smart contracts on the blockchain. Following a successful registration process, the blockchain system allocates a unique anonymous identity (ID) and generates associated certificates (Cert), public keys (PK), private keys (SK), and wallet addresses (WA) to each node. These certificates play a fundamental role in user identity verification, and the mapping list (ID, Cert, PK, and WA) is securely stored within the account pool. Moreover, these data are meticulously cataloged in a comprehensive global information repository, which is under vigilant maintenance and monitoring by the control node.

Subsequently, the controller conducts periodic data collection on idle resources and sharing preferences from ROs, and stores these data in a dedicated database. Upon instantiating a new VNF, the IP utilizes real-time data from the database. This involves employing both the utility game model and the greedy matching algorithm. The goal is to efficiently match and select the most advantageous resource-sharing scheme in collaboration with the RO. The chosen scheme is then conveyed to the smart contract.

Finally, the secure allocation of shared resources is achieved through a blockchain-based encrypted access control approach. This process primarily involves key generation, resource address encryption, access policy concealment, and resource address decryption.

In the phase of key generation, the secret key (SK) is generated through the utilization of the key generation algorithm. This algorithm requires the public key, master key, and the attribute set that is linked to the resource demand collection as its input.

Moving to the resource address encryption phase, the RO initially assigns unique IDs to each shared resource. By employing a hash function, the corresponding indices (index) are derived and subsequently stored on the blockchain via a smart contract. The contract address (addr) is then communicated to the IP. Following this process, the RO independently encrypts the resource addresses and access policies, resulting in the creation of two distinct ciphertexts: encrypted address (ADC) and access policy (ACC). The aforementioned ciphertexts are securely stored on the blockchain.

In the access policy concealment phase, a Bloom Filter is employed to obscure the access policies. This process yields the creation of an Adaptive Bloom Filter (ABF), which is then stored on the blockchain, while the previous policy function is eliminated.

During the decryption phase, the IV initiates the calculation of the index and ABF associated with the shared resource by using the addr obtained from the IP. Access legitimacy is verified through a smart contract by facilitating the reconstruction of the policy function. The process of reconstruction facilitates the retrieval of the ADC and ACC ciphertexts, ultimately leading to the execution of the decryption algorithm.

## 4. Resource Sharing Game Model

In the initial phase of VNF instantiation, the VNF sends a resource request to the IP, represented as the vector $R=\{r_{1},r_{2},\cdots ,r_{n}\}$, where each element $r_{i}$ is a non-negative ($r_{i}\geq 0$). The decision-making process pertaining to the allocation can be approached in two ways. The IP assesses whether to allocate dedicated resources exclusively for the VNF or to adopt a resource-sharing strategy with ROs. The determination of this decision depends on the current availability of free resources across servers and the potential profitability associated with resource sharing with ROs. Within this context, it is assumed that there are *m* ROs who have the capability to share a time slice in which resource *i* is available. For clarity and ease of reference, essential notations pertinent to this section are conveniently presented in Table 1.

### 4.1. Assumptions

Resource allocation for a given resource, denoted as *i*, encompasses two distinct modes: exclusive allocation or sharing with a maximum of one VNF without any further subdivision.

In contrast, various resources, labeled as *i* and *j*, exhibit the flexibility to be concurrently shared with a common VNF or independently allocated to distinct VNFs.

### 4.2. Infrastructure Provider Utility Model

Upon receiving the resource demand set *R*, if the IP decides not to implement a resource-sharing strategy and instead opts to allocate exclusive resources from the resource pool for the upcoming VNF deployment, and the cost associated with each resource *i* can be mathematically expressed as follows:

(1) $$C_{P}^{i}=\mu_{i}r_{i}$$

where $\mu_{i}$ denotes the unit cost of resource *i*.

The aggregate cost of consumed resources is computed as

(2) $$C_{P}=\sum_{i=1}^{n}\mu_{i}r_{i}$$

In the scenario where the IP implements a sharing approach, whereby a portion of the resource requirements needed to instantiate a VNF is allocated to ROs based on their specific interests, the formulation of the utility function for deploying resource *i* to $RO_{j}$ can be expressed as follows:

(3) $$U_{i}^{j}=\left(1-\sigma_{j}\right)\upsilon_{i}\ln\left(1+r_{i}^{j}\right)$$

(4) $$r_{i}\leq F_{i}^{j}$$

(5) $$0<\sigma_{i}<1$$

(6) $$\upsilon_{i}\geq 1$$

(7) $$r_{i}^{j}=\left\{\begin{array}{cc} r_{i} & RS_{j}^{i}\\ 0 & else \end{array}\right.$$

Here, $\sigma_{j}$ represents the degree of relevance between the IV and $RO_{j}$. A higher value of $\sigma_{j}$ indicates a more robust alignment of business interests between the two VNFs, thereby increasing the likelihood of conflicts in network service timing and spatial usage. As a corollary, this leads to a diminished utility value. Therefore, it is imperative for the IP to make an effort in selecting the RO whose business profiles significantly vary from the instantiated VNF, thereby facilitating efficient resource sharing. The term $F_{i}^{j}$ denotes the quantity of the available resource *i* within $RO_{j}$ during the specified time period. Meanwhile, $\upsilon_{i}$ signifies the utility level of resource *i*. Importantly, the relationship between $\upsilon_{i}$ and the remaining quantity of resource *i* in the IP’s current servers is negative. In other words, as the quantity of resource *i* diminishes in the servers, the IP’s incentive to engage in resource sharing with ROs increases, resulting in a higher sharing utility. Additionally, $RS_{j}^{i}$ signifies the allocation of resource *i* through the sharing mechanism with $RO_{j}$.

To articulate the inverse relationship between the available resources and their influence on the IP utility function, we delineate the computation methodology for $\upsilon_{i}$, as follows

(8) $$\upsilon_{i}=\left\{\begin{array}{c} \frac{r_{i}^{I}-r_{i}}{r_{i}^{I}},r_{i}\leq r_{i}^{I}\\ \frac{r_{i}}{r_{i}-r_{i}^{I}},r_{i}>r_{i}^{I} \end{array}\right.$$

where $r_{i}^{I}$ represents the remaining quantity of resource *i* in the IP.

The model ensures that the utility function of the IP is a non-negative, strictly concave, and twice continuously differentiable increasing function. Additionally, in cases where resource *i* is not participating in the sharing process, the utility value is assigned a value of zero.

In the scenario where the $RO_{j}$ sets the unit price for resource *i* as $\rho_{i}^{j}$, and the IP sets the unit retail price for resource *i* as $\kappa_{i}$, the sum resulting profit for the IP is

(9) $$P_{I}=\sum_{j=1}^{m}\sum_{i=1}^{n}\kappa_{i}r_{i}+U_{i}^{j}-\rho_{i}^{j}r_{i}^{j}-\mu_{i}r_{i}^{0}$$

(10) $$r_{i}^{0}=\left\{\begin{array}{cc} r_{i} & NRS_{i}\\ 0 & else \end{array}\right.$$

where $NRS_{i}$ stands for resource *i* and is allocated by the exclusive resource.

The objective of the IP is to optimize profit, that is

(11) $$\max P_{I}=\sum_{j=1}^{m}\sum_{i=1}^{n}\kappa_{i}r_{i}+\left(1-\sigma_{j}\right)\upsilon_{i}\ln\left(1+r_{i}^{j}\right)-\rho_{i}^{j}r_{i}^{j}-\mu_{i}r_{i}^{0}$$

### 4.3. Resource Owner Utility Model

Within NFV, ROs manifest their inclination to allocate presently dormant resources to other VNFs. This endeavor is pursued with the overarching objective of maximizing the revenue potential during periods when available resources would otherwise remain unutilized. However, it is crucial for ROs to judiciously contemplate the concomitant costs entailed in the allocation of their resources. The cost function pertaining to resource *i* within the purview of $RO_{j}$ is devised as

(12) $$C_{i}^{j}=\sigma_{j}\lambda_{i}^{j}r_{i}^{j}$$

where $\lambda_{i}^{j}$ signifies the cost level of $RO_{j}$ for resource *i*. As mentioned earlier, the parameter =$\sigma_{j}$ reflects the degree of correlation between the IV and the RO. For analogous reasons, a higher correlation enhances the likelihood of conflicts in the timing of network services and the utilization of space. Hence, the cost associated with shared services is positively proportional to the value of $\sigma_{j}$.

Therefore, it can be concluded that the profit of the $RO_{j}$ is

(13) $$P_{R}^{j}=\sum_{i=1}^{n}\rho_{i}^{j}r_{i}^{j}-C_{i}^{j}$$

For the $RO_{j}$, the objective is also to maximize profit, that is

(14) $$\max P_{R}^{j}=\sum_{i=1}^{n}\rho_{i}^{j}r_{i}^{j}-\sigma_{j}\lambda_{i}^{j}r_{i}^{j}$$

## 5. Greedy Matching Algorithm

Preliminary work: ROs engage in creating a smart contract by utilizing their existing resources and pricing strategy. This smart contract is subsequently deployed on the Ethereum, resulting in the acquisition of a unique contract address denoted as ‘addr’. Concurrently, each shared resource is assigned an ID by the RO, and its corresponding index is determined by applying a hash function. Following this, the ROs convey both the contract address ‘addr’ and the generated IDs to the IP. This enhances the ability of the IP to access up-to-date information regarding the availability of shared resources.

The proposed resource sharing model introduces a game-theoretic problem. From the perspective of ROs, the strategy to maximize profits entails setting higher prices for unit resources. However, if the unit resource price is set too high, it may have the unintended consequence of reducing the revenue of the IP and potentially discouraging the IP from selecting the RO as a partner for resource sharing. Notably, the utility function of the IP and the cost function of the RO are both influenced by the correlation parameter $\sigma_{j}$. As demonstrated in Equations (11) and (14), a decrease in the value of $\sigma_{j}$ enhances the probability of both parties attaining optimal earnings simultaneously. Consequently, ROs with a lower correlation to the IV in the current system may intentionally set a higher unit resource price, for engaging in a strategic competition among multiple ROs.

From an alternative perspective, the IP is assigned the responsibility of disseminating the resource requirements of the IV among separate ROs for either resource sharing or the allocation of exclusive resources. Consequently, the issue of resource sharing encompasses a scenario where ROs and the resource demand vector *R* need to be matched.

Suppose resource *i* is deployed into the shared resources of $RO_{j}$; at this point, the revenue for the IP with respect to resource *i* is denoted as

(15) $$P_{I}^{i}=\kappa_{i}r_{i}+\left(1-\sigma_{j}\right)\upsilon_{i}\ln\left(1+r_{i}^{j}\right)-\rho_{i}^{j}r_{i}^{j}$$

**Figure 3:**
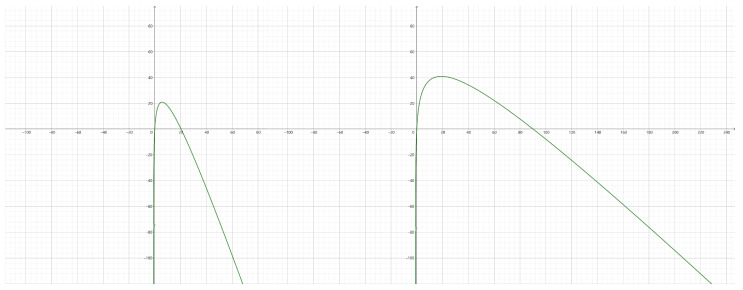
Plots of $P_{j}^{i}$ in two different sets of parameters.

From the plots of this function in Figure 3, it can be observed that there exists a peak revenue point. Additionally, for a given resource *i*, the implementation of different pricing strategies by the RO can result in distinct peak revenue points and corresponding resource quantities. These variations are influenced by factors such as the correlation and utility level, which are predetermined parameters. Furthermore, specific resource quantity requirements are essential to ensure a positive revenue. Through an analysis of this function, it can be deduced that the optimal resource quantity that maximizes revenue is denoted as

(16) $$r_{i}^{max}=\frac{\upsilon_{i}\left(1-\sigma_{j}\right)}{\rho_{i}^{j}}-1$$

and the maximum benefit is

(17) $$P_{I}^{imax}=\rho_{i}^{j}+\upsilon_{i}\left(\sigma_{j}+\ln\left(\frac{\upsilon_{i}\left(1-\sigma_{j}\right)}{\rho_{i}^{j}}\right)\left(1-\sigma_{j}\right)-1\right)+\kappa_{i}r_{i}$$

In the context of VNF instantiation, where the quantity of resource requests $r_{1}^{j}$ remains constant, the IP must make judicious decisions among various ROs to optimize outcomes, aiming to closely approximate the extremum of the revenue. From the RO’s perspective, the ongoing game constitutes an information-symmetric scenario due to the storage of information in the blockchain. The RO possesses comprehensive knowledge of all resource demand situations. Consequently, in the pursuit of maximizing their earnings, each RO will strategically adjust the unit price of resources to align with the objective of maximizing profit for the target resource, while also attracting the attention of the infrastructure provider.

The matching process for each shared resource $r_{i}$ consists of two distinct stages: the price competition stage among ROs and the IP decision stage.

During the stage of price competition among ROs, a game of pricing strategy unfolds. Each RO determines the price $\rho_{i}^{j}$ for each resource based on their utility parameter $\sigma_{j}$ and the pricing strategies employed by other ROs. The main goal is to attract the interest of the IP and optimize financial gains. It is assumed that each RO acts rationally and possesses access to the utility function parameters of other ROs through information conveyed in smart contracts. Consequently, this scenario establishes a game of perfect information price competition.

In the RO bidding stage, where all ROs participate as players, the strategy for $RO_{j}$ is to choose a price $\rho_{i}^{j}$ from the feasible set $\left[P_{low}^{j},P_{high}^{j}\right]$. Their objective is to select $\rho_{i}^{j}$ that satisfies

(18) $$\arg\max P_{i}^{j}=\rho_{i}^{j}r_{i}^{j}-\sigma_{j}\lambda_{j}r_{i}^{j}$$

s.t.

(19) $$\rho_{i}^{j}\subseteq \left[P_{low}^{j},P_{high}^{j}\right]$$

(20) $$0\leq P_{low}^{j}\leq r_{i}^{j}\leq P_{high}^{j}$$

Here, $P_{low}^{j}$ and $P_{high}^{j}$ denote the two points intersecting the *x*-axis in Figure 3. These points signify optimal choices for $\rho_{i}^{j}$. Their selection ensures that the demand for resources falls within a range where the IP revenue remains positive.

In the IP decision stage, subsequent to obtaining a price list from all ROs for resource *i*, the IP needs to decide whether to allocate exclusive resources to the IV or opt for resource sharing with a specific RO. The objective is to select $NS_{i}^{j}$ or NRS such that

(21) $$\arg\max P_{I}^{i}=\kappa_{i}r_{i}+\left(1-\sigma_{j}\right)\upsilon_{i}\ln\left(1+r_{i}^{j}\right)-\rho_{i}^{j}r_{i}^{j}-\mu_{i}r_{i}^{0}$$

Through the above analysis of the game model, we devised a greedy matching algorithm in Algorithm 1.
**Algorithm 1** Greedy Matching Algorithm**Input**:          Resource demand vector *R*;          Unit cost of resource *i* for IP $\mu_{i}$;          The degree of relevance between IV and ROs $\sigma_{j}$(j=1,2,⋯,m);          The cost level of Rs $\lambda_{j}\;\left(j=1,2,\cdots ,m\right)$;**Output**:          Resource Allocation plan for IV $r_{i}^{j}$, $r_{i}^{0}$(j=1,2,⋯,m)(i=1,2,⋯,n);          The profit of IP $P_{I}$;          The profit of ROs $P_{R}^{j}\;\left(j=1,2,\cdots ,m\right)$;**Begin**01:   **Initialize**          The utility level of resource $\upsilon_{i}$;          The free amount of resource *i* in $RO_{j}$$F_{i}^{j}$;          The unit retail price for resource *i* of IP $\kappa_{i}$;02:   **FOR** Resource *i* in demand vector *R*03:         ROs engage in pricing strategy games according to Equations (18)–(20),     resulting in price sequences ρ for resource *i*;04:         Calculate IP’s profit $P_{I}^{i}$ for each RO by Equation (21);05:         Sort $P_{I}^{i}$;06:         IP selects the largest $P_{I}^{i}$ for a decision or allocates exclusive resources for IV07:         Update $F_{i}^{j}$, the free amount of resource *i* in $RO_{j}$08:   **ENDFOR****End**

## 6. Blockchain-Based Encrypted Access Control Approach

Upon achieving a match in resource sharing, ROs and IVs participate in encrypted resource allocation and access control, which is facilitated through blockchain coordination.

### 6.1. Bilinear Mapping

In the context of cryptographic operations, a pairing, denoted as $e:G_{0}\times G_{0}\to G_{1}$, is a fundamental bilinear mapping. In this representation, $G_{0}$ and $G_{1}$ refer to cyclic groups of prime order *p*, with *g* serving as a generator for $G_{0}$.

The pairing operation *e* is characterized by key properties:

**Bilinearity**: For any $P,\;Q\in G_{0}$ and non-zero $a,\;b\in Z_{p}$, it holds that $e\left(P^{a},\;Q^{b}\right)=e{\left(P\;,Q\right)}^{ab}$.
**Non-degeneracy**: The property of non-degeneracy ensures that e(g, g)≠1, particularly when *g* operates as a generator of $G_{0}$.
**Computability**: There exists an algorithm available that efficiently computes this mapping within a polynomial time complexity.

### 6.2. Linear Secret Sharing Scheme (LSSS)

In the context of LSSS [37], let *U* denote the attribute domain, and *p* stand as a prime number. For every access structure *M* defined on *U*, *M* is essentially an *r* by *n* matrix over the field $Z_{p}$. The rows of this matrix *M* are associated with mappings to ρ(1,2,…,i). Here, a secret value *s* ($s\in Z_{p}$) and a set of random numbers $l_{1},l_{2},\ldots ,l_{n}\in Z_{p}$ collectively compose the vector $\vec{v}=\left(s,l_{2},l_{3},\ldots ,l_{n}\right)$, and its transpose is represented as $\vec{v^{\prime }}={\vec{v}}^{T}$. Consequently, the product $M\vec{v^{\prime }}$ yields *r* secret shares denoted as $\omega_{i}={\left(M\vec{v^{\prime }}\right)}_{i}$, each corresponding to the secret share held by ρ(i).

In terms of Linear Reconstruction, the focus is on an authorized attribute set *S*, where I={i:ρ(i)∈S}⊆{1,2,…,r}. In this context, elements $c_{i},c_{j}\in A$ are introduced, with the stipulation that for any B,C∈A where B⊆C, it holds true that C∈A. This particular characteristic defines *A* as a monotonic access structure. For the purposes of this paper, we specifically emphasize monotonic access structures. In the realm of Attribute-Based Encryption (ABE), the traditional roles of entities are replaced by attributes, thus integrating authorized attribute sets within the broader access structure *A*.

### 6.3. Algorithm Steps

Step 1 Initialization:

Given a security parameter λ, the initialization algorithm chooses two cyclic groups $G_{0}$ and $G_{1}$ of prime order *p*, Additionally, it designates a generator *g* for $G_{0}$ and defines a bilinear mapping *e* with the functionality $e:G_{0}\times G_{0}\to G_{1}$. The process of selecting and mapping groups is accomplished by utilizing the group generator algorithm. Furthermore, random elements $h,k,q\in G_{0}$ and $\alpha ,\beta \in Z_{p}$ are generated. The public key PK is then computed as

$$PK=\{G_{0},p,g^{\alpha },g^{\beta },h,k,e{\left(g,g\right)}^{\alpha }\}$$

and the master key as MSK={α,s,t}.

The process is initiated by the RO through the creation and deployment of a smart contract on the blockchain. This results in the acquisition of a designated contract address, which is denoted as addr. Subsequent to this step, the RO allocates a distinctive identifier, denoted as ID, and designed for the upcoming configurations of shared resources. Following the identifier assignment, the Hash method is employed to calculate an index, and both the contract address (addr) and the identifier (ID) are securely stored within the domain of the IP. Lastly, the computed index is transmitted to the blockchain through the smart contract.

Step 2 Encryption:

The attribute set of the resource *i* to be shared by RO is recorded as $Rs=att_{1},att_{2},att_{n}$. By selecting *u* randomly from the set of $u_{1},u_{2},\ldots ,u_{m}\in Z_{p}$, the following elements are computed:

$$D=g^{\beta }q^{u}$$

$$H=g^{u}$$

$$X_{j,1}={\left(h^{att_{j}}g^{\beta }\right)}^{u_{i}}k^{-u}$$

$$X_{j,2}=g^{u_{j}}$$

These calculations result in the generation of a private key as

$$SK=\{S,D,H,X_{j1},X_{j2}\}$$

Subsequently, the system selects *w* randomly from $Z_{p}$ and computes $key=e{\left(g,g\right)}^{\alpha w}$ by utilizing the PK and the ID of the resource. For each attribute in $Z_{p}$, denoted as $att_{i}$ and letting $\theta_{i}$ be a share of *w*, we can compute

$$Y_{i,1}=qk^{\theta_{i}}$$

$$Y_{i,2}={\left(h^{att_{i}}g^{\beta }\right)}^{-\theta_{i}}$$

$$Y_{i,3}=g^{\alpha \theta_{i}}$$

These elements, in combination with the selected parameters, give rise to a partial ciphertext represented as:

$$CTpre=\{key,g^{w},w,att_{i},s_{i},Y_{i,1},Y_{i,2},Y_{i,3}\}$$

The RO then employs an attribute-based encryption algorithm to encrypt the resource key RK. By utilizing the public key PK, the resource key RK, the LSSS-based access structure (M,ρ), and the $CT_{pre}$, the ciphertext CT is generated. In the access structure (M,ρ), *M* is an r×n matrix, and the computation of $Y_{i,4}$ is carried out as

$$CT=(\left(M,\rho \right),g^{w},Y,Y_{i,1},Y_{i,2},Y_{i,3},Y_{i,4})$$

Then, the RO maps the CT to the index and uploads this mapping to the blockchain. The RO utilizes a smart contract to define the validity access time for the CT.

Within the framework of this LSSS-based CP-ABE scheme, an attribute Bloom filter (ABF) is established through the following series of steps:

(1) The RO extracts the attribute set RS from the access policy defined in the access structure (M,ρ). An element in the ABF, denoted as *e*, is structured as $e=(r||att_{i})$, where *r* signifies the row number of the matrix *M*, and $att_{i}$ represents one of the attributes. These components are transformed into bit strings of lengths $L_{rownum}$ and $L_{att}$, respectively.
(2) The $L_{rownum}$ bit string and $L_{att}$ bit string are combined into a λ-bit string. An element $s=(i||att_{s})$ is introduced to the ABF, where *s* constitutes a secret share value. To achieve this, n−1λ bit strings $l_{1},l_{2},\ldots ,l_{n-1}$ are randomly obtained, and $l_{n}=l_{1}⊕l_{2}⊕\ldots ⊕l_{n-1}⊕s$ is computed through secret sharing.
(3) Hash functions are applied to $att_{s}$ of element *s* in order to derive index positions for each share value within the ABF as $h_{1}\left(atts\right),h_{2}\left(atts\right),\cdots ,h_{n}\left(atts\right)$.
(4) The RO proceeds to store each shared value at the corresponding hash index location. Subsequently, the RO uploads both the ABF and the access matrix *M* to the IP.

Step 3 Decryption:

The IV initiates the process by obtaining the Resource Identifier ID, the address of the smart contract, and the encrypted data stored by the RO on the IP. The IV then proceeds with the following steps:

1. ID Verification and Resource Existence Check: The IV hashes the received ID and executes the smart contract to validate the existence of the requested resource on the blockchain. If the resource cannot be located, the algorithm terminates.
2. Ciphertext Access Time Check: Upon obtaining the ciphertext’s ID through Algorithm 2, the IV first checks whether it falls within the valid access time period. If access is not granted, termination occurs. Otherwise, the user proceeds to acquire the ciphertext CT of the Resource Key RK.
3. Attribute-Based Policy Verification: Before decrypting the ciphertext, the IV must ensure that its attributes satisfy the access policy. This involves restoring the policy function ρ.
4. The reconstruction of the policy function ρ from the Attribute Bloom Filter (ABF) is performed through the following steps:
  (1) Utilize *n* hash functions to hash the attributes
    $$h_{1}\left(att_{s}\right),h_{2}\left(att_{s}\right),\cdots ,h_{n}\left(att_{s}\right)$$
  (2) Obtain the corresponding strings through position indexes;
  (3) Calculate the shared value *s* and output the corresponding string:
    $$s=l_{1}⊕l_{2}⊕\cdots ⊕l_{n}$$
  (4) Represent *s* as s=(r||att), and compare att with $att_{s}$. A match signifies the presence of the attribute in the ABF, while att denotes the attribute’s position within the access matrix *M*. A mismatch indicates that the attribute is absent in the ABF.
  (5) Upon the successful restoration of the access structure (M,ρ), the IV proceeds with the decryption process.
5. Resource Key Retrieval: With the reconstructed access structure (M,ρ), the IV is able to decrypt the ciphertext CT to obtain the Resource Key (RK). The computation involves verifying that the IV’s attributes align with the access policy and calculating RK based on authorized attribute sets and shared values.

**Algorithm 2** IV Gets CT
**Input**: Resource ID
**Output**: Cipher text CT

**Begin**

01:   index = hash(ID);
02:   **IF** index = null
03:         Return error;
04:   **ELSE**
05:         Mapping(index⇒ CT.available_time)
06:         **IF** Runing time is expired
07:                Return error;
08:         **ELSE**
09:                Mapping(index⇒ RO.available_time)
11:         **IF** Sharing time is expired
12:                Return error;
13:         **ELSE**
14:                Mapping(index⇒CT)
15:   **ENDIF**

**End**




## 7. Simulation Results

In this section, we conduct extensive simulation experiments to evaluate the performance of the proposed mechanism.

### 7.1. Simulation Setup

In this section, we delineate the simulation setup employed for the assessment of the proposed system, which relies on the Open Network Automation Platform (ONAP) [38]. Additionally, we elucidate the blockchain module crafted utilizing the Ethereum platform [39] and implemented through the Solidity smart contract language [40].

1. Experimental Environment

The simulation experiments were carried out in a controlled environment, utilizing specific software and hardware configurations.

**Figure 4:**
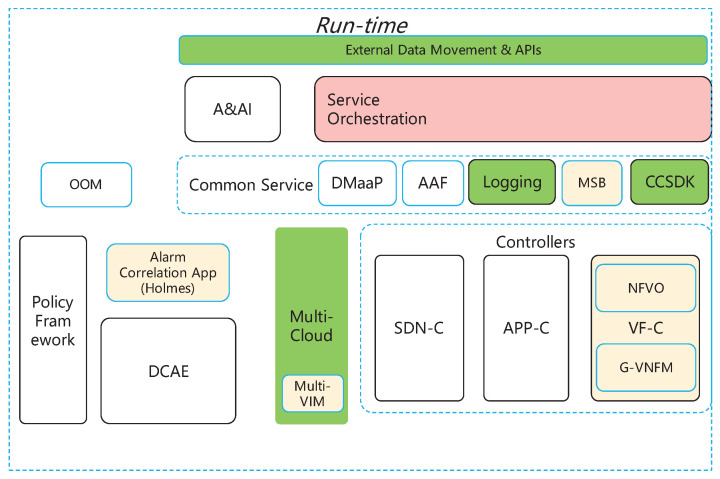
Structure of Run-time in ONAP.

Software:

- ONAP Run-time: We employed the Run-time module of the ONAP framework to facilitate VNF instantiation. The Run-time model offers the essential interfaces and standards for VNF management. In our study, we specifically modify the code of the Virtual Function Controller (VF-C) in order to implement our framework. The structure of the Run-time is depicted in Figure 4.
- Ethereum Platform: We deployed the blockchain module on the Ethereum platform, which served as the underlying infrastructure for executing the smart contracts.
- Solidity: We used Solidity, a high-level language for coding smart contracts, to develop the necessary smart contracts for implementing the blockchain module.

Hardware:

- Processor: Intel Xeon(R) Gold 6238R (DELL PowerEdge R740, made in Shenzhen, China);
- Memory: 200 GB RAM;
- Storage: 1 TB HDD.

2. Simulation Scenarios

We conducted a series of simulation scenarios. The key parameters and variables considered in our simulations are as follows:

We varied the number of ROs and the length of the resource requirement vector (*R*). Scenarios were explored across RO counts from 1 to 50, and resource requirement vector lengths ranging from 1 to 10. The quantity of resource *i* required by the IV, denoted as $r_{i}$, underwent investigation across a range of values from 1 to 50. We evaluated the degree of relevance ($\sigma_{j}$) between the IV and each $RO_{j}$, as well as the cost level of $RO_{j}$ for resource *i* ($\lambda_{i}^{j}$). Both $\sigma_{j}$ and $\lambda_{i}^{j}$ were assumed to follow a uniform distribution, with values ranging from 0.1 to 0.9, respectively. Additionally, the unit cost of resource *i* for the IP ($\mu_{i}$) was randomly set between 100 and 300, while the unit retail price for resource *i* of IP ($\kappa_{i}$) was randomly set to be 25% to 35% higher than $\mu_{i}$. For convenience, a summary of the parameters and their respective value ranges is presented in Table 2.

3. Evaluation Metrics

To assess the effectiveness and efficiency of our proposed system, we employed the following evaluation metrics:

Social utility: The social utility, denoted as *S*, is a measure used to assess the efficiency of resource allocation by considering the combined revenue generated by both the RO and the IP. The computation of this social utility involves assessing the disparity between the utility of resource sharing provided by the IP and the cost of resource contribution borne by the RO. Mathematically, it is expressed as

(22) $$S=U_{i}^{j}-c_{j}^{i}=\left(1-\sigma_{j}\right)\upsilon_{i}\ln\left(1+r_{i}^{j}\right)-\sigma_{j}\lambda_{j}r_{i}^{j}$$

Running time of matching algorithm: To gauge the algorithm’s performance, we conducted extensive runtime tests under different scenarios. These tests provided insights into the algorithm’s computational efficiency, and comparative analyses were conducted with the results obtained from alternative algorithms.

Performance of access control: We conducted tests primarily aimed at comparing the efficiency of the proposed algorithm in terms of encryption and decryption with that of other algorithms in the same category.

### 7.2. Performance of Greddy Matching Algorithm

#### 7.2.1. Validity Test of the Algorithm

**Figure 5:**
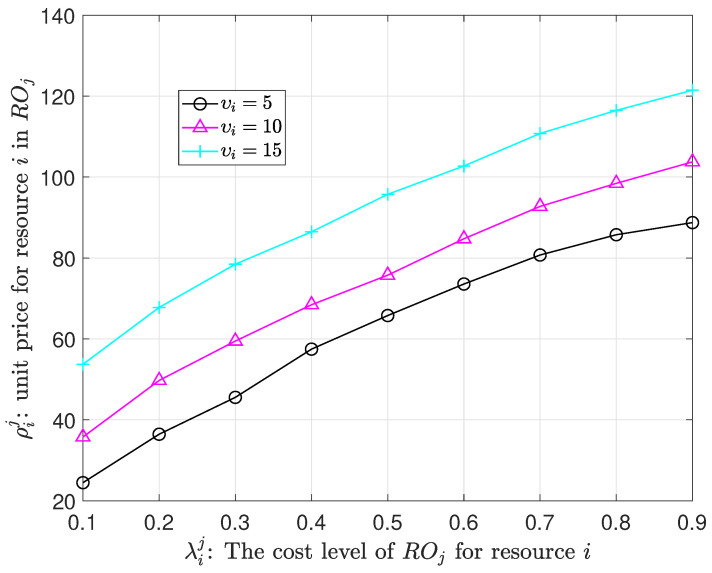
RO’s pricing strategy under different utility parameters.

Figure 5 illustrates the pricing dynamics of RO for shared resources concerning the variations in the cost level of $RO_{j}$ for resource *i* under different utility levels of $\upsilon_{i}$.

It becomes apparent that a relatively high value of $\upsilon_{i}$, which indicates resource scarcity within the IP, results in an increased tendency towards resource sharing. Consequently, this tendency enables ROs to set higher prices for their shared resources, ultimately enhancing their profitability.

Furthermore, within the proposed framework, the pricing strategy employed by ROs for shared resources is positively correlated with the growth of $\lambda_{i}^{j}$. In instances where the cost level of $RO_{j}$ for resource *i* is low, ROs opt to set lower prices in order to attract a greater demand. Conversely, when faced with higher values of $\lambda_{i}^{j}$, ROs find themselves dealing with more substantial costs related to resource sharing, compelling them to institute higher prices for their shared resources.

This strategic pricing mechanism efficiently captures the dynamic characteristics of the resource-sharing ecosystem. The experimental results underscore the validity of the proposed mechanism and algorithm, which foster a healthy competitive environment among ROs and enable efficient negotiation between ROs and the IP.

#### 7.2.2. Algorithm Performance Comparison

The resource sharing model introduced in this research can be viewed as a specialized variant of the Stackelberg game model.

The Stackelberg game is a strategic interaction model within game theory that involves players with asymmetric positions. In this model, one player assumes the role of the leader, while the others become followers [41]. The leader takes the initiative in decision making, and the followers carefully observe these decisions before formulating their own choices. This distinctive feature of sequential decision making differentiates the Stackelberg game from simultaneous-move games.

Typically, the leader’s objective is to maximize its own utility or payoff, while considering the anticipated reactions of the followers. Concurrently, the followers strive to optimize their outcomes based on the leader’s decisions. The advantage of a leader resides in its capacity to exert influence over the ultimate outcome by strategically shaping choices that consider the reactions of the followers.

Stackelberg games are solved using diverse methods based on game characteristics and players’ information. Common approaches include Mathematical Programming (linear or nonlinear optimization), Nash Equilibrium Analysis, Dynamic Programming for sequential decisions, Simulation and Computational Methods, Game Tree Analysis for visualizing decisions, Optimal Control Theory for dynamic games, and considering Learning and Adaptive Strategies for realistic behaviors.

Particularly, the diagonalization method serves as a valuable tool for resolving Stackelberg games by transforming the initial non-diagonal structure of the game into a diagonal format. This transformation facilitates independent decision-making for both the leader and the follower, simplifying the analytical process. The diagonalization method is notably efficient in computing the Nash equilibrium and determining strategies for both players in a Stackelberg game.

To evaluate the performance and efficacy of the proposed greedy-based matching algorithm, a comparative analysis was carried out in comparison to the diagonalization method. This comparison aimed to assess the optimization capabilities of the two algorithms in terms of achieving optimal social utility and algorithm performance.

For the sake of brevity, the algorithm introduced in this paper is referred as GA, while the diagonalization method is denoted as DA.

**Figure 6:**
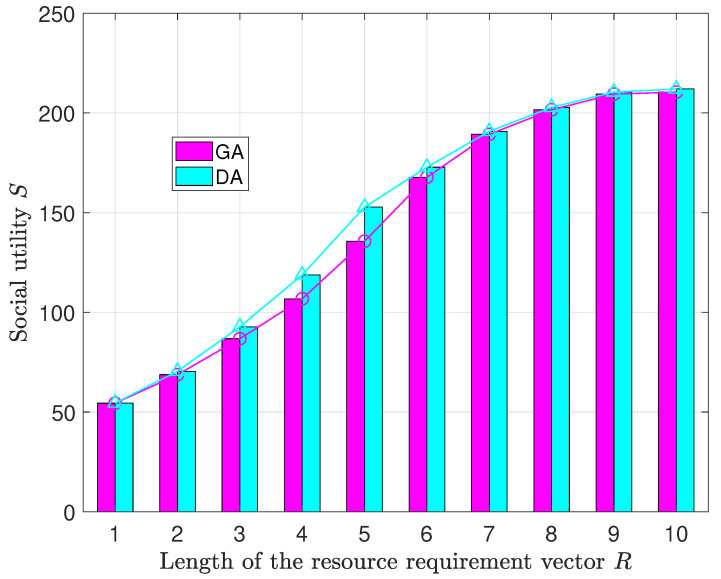
The relationship between social utility and the number of resource types.

Figure 6 delineates the dynamics of social utility (*S*) in the context of both GA and DA. The examination investigates the impact of varying the length of the resource requirement vector (*R*), while keeping a constant number of 35 ROs.

The observations consistently underscore a discernible pattern: in both algorithms, an augmentation in the diversity of resource demands, as indicated by the length of *R*, corresponds to an increase in social utility. This increasing trend is propelled by a heightened interest in resource sharing among ROs, particularly when the available resources of IPs face constraints due to the proliferation of resource types ($\upsilon_{i}$ becoming larger). ROs are more incentivized to contribute their dormant resources.

As the demand for resources increases, the rate of growth in social utility gradually decelerates. When the length of the resource demand vector *R* exceeds 9, the rate of change in social utility approaches zero. This is because the variety of resource demands increases to a significant extent, resulting in a scarcity of idle resources for ROs. Consequently, a saturation point is reached where the rate of social utility growth decelerates.

Furthermore, the results indicate that the proposed algorithm GA has slightly lower results than the diagonalization method DA in terms of social utility. The minimum difference occurs when the length of the resource demand vector is *R* 1, with a difference of 0.11. The maximum difference occurs when the length of the resource demand vector *R* is 5, with a difference of 17.14. This difference arises from the precision of DA, which has the capability to achieve optimal objectives. In contrast, GA relies on a greedy strategy, which offers a heuristic approach that does not fully consider the overall global social utility.

**Figure 7:**
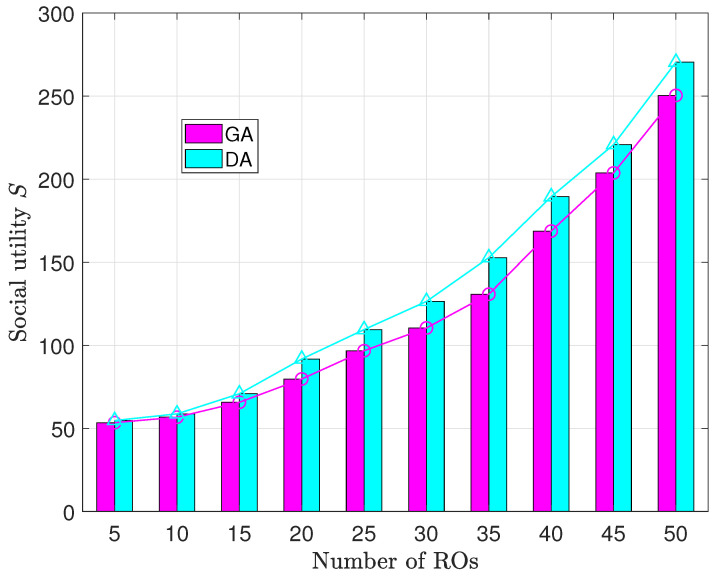
The relationship between social utility and the number of ROs.

The experiments illustrated in Figure 7 are conducted with a constant resource requirement vector length *R* of 5. The objective is to examine how social utility *S* changes as the quantity of ROs varies.

In both algorithms being analyzed, it is evident that there is a positive correlation between the number of ROs and the increase in social utility. However, the trend diverges from Figure 6. Here, the rate of social utility growth intensifies with a larger RO pool. When the number of ROs increases from 5 to 10, the growth rates of the social utility of GA and DA are 0.55 and 0.802, respectively. Nevertheless, as the number of ROs increases from 45 to 50, the growth rates of social utility for GA and DA are 9.332 and 9.928, respectively. This phenomenon can be attributed to the escalating deployment of VNFs in conjunction with a rising number of ROs. Moreover, this trend indicates a reduction in the availability of exclusive resources for IP, resulting in increased values of $\upsilon_{i}$. Subsequently, the increased scarcity of limited resources necessitates that IP prioritize the allocation of shared resources for newly requested instances of VNFs. As a result, there is a noticeable intensification in the rate of the growth of social utility.

Consistent with previous observations, GA lags marginally behind DA concerning social utility. This outcome can be attributed to the characteristics of GA, for the same reasons as previously mentioned.

Another performance evaluation of the proposed algorithms focuses on runtime, specifically in relation to the length of the Resource Requirement Vector *R* and the number of ROs. This analysis is aimed at uncovering how these variables impact the efficiency of the algorithms.

**Figure 8:**
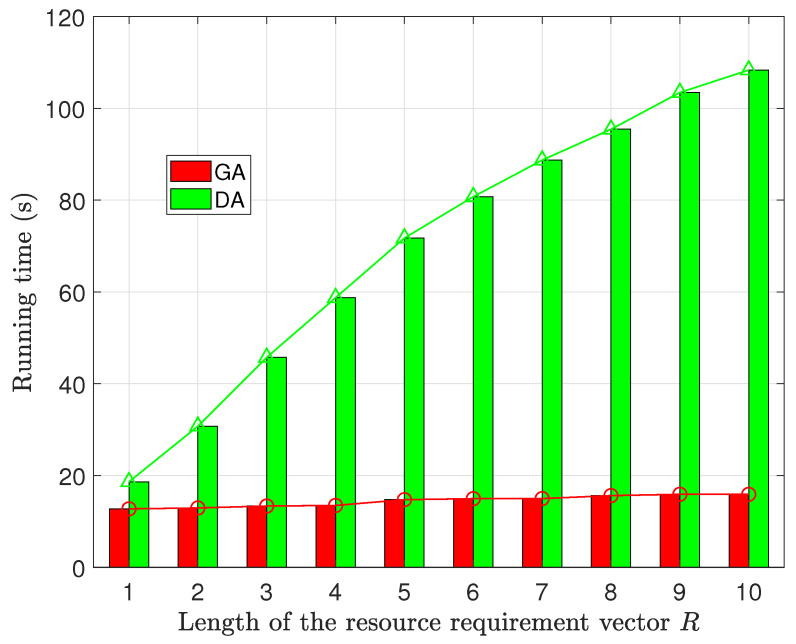
The relationship between Running time and the number of resource types.

**Figure 9:**
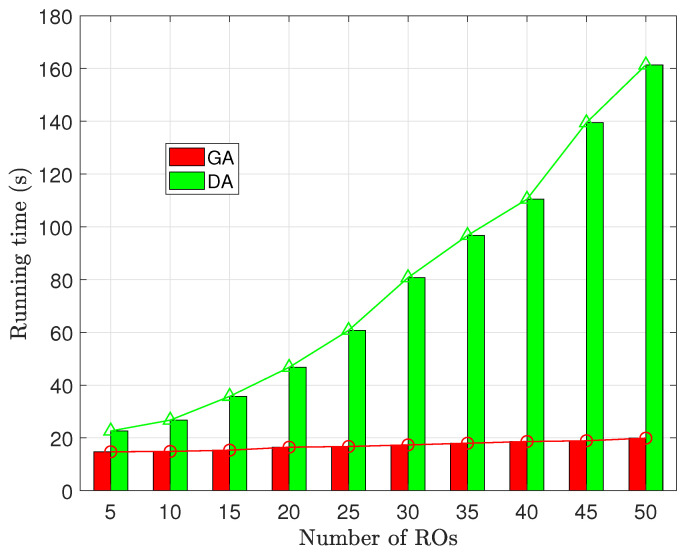
The relationship between Running time and the number of ROs.

The results, presented in Figure 8 and Figure 9, show that GA displays a remarkable stability in its runtime, with minimal sensitivity to variations in both *R* and the number of ROs. This suggests that GA consistently maintained high efficiency under diverse scenarios.

In contrast, the runtime of DA significantly increases as both the length of the resource requirement vector and the number of ROs increase. The longest runtime for DA is 8.1 times that of GA. This behavior is attributed to the DA’s iterative solving approach, which does not guarantee a predictable convergence speed. When confronted with a larger number of participants, such as multiple leaders or followers in the game model, the solving process of DA becomes less efficient, leading to a longer runtime.

Furthermore, it can be observed that the runtime of DA follows a similar trend to the growth rate of social utility. This occurs because the increase in social utility is driven by high-quality resource sharing, which necessitates more iterations in DA’s solving process. As a result, there is a similar upward trend in runtime as social utility increases.

The conducted experiments reveal that our proposed greedy matching strategy, although falling short of attaining the optimal social utility inherent in the standard Stackelberg game solution, demonstrates significant efficiency that remains largely unaffected by variations in the problem scale. Moreover, the outcomes produced by our algorithm exhibit only marginal deviation from the optimal solution. This characteristic renders our approach well-rounded and efficient.

### 7.3. Performance of Blockchain-Based Encrypted Access Control Approach

#### 7.3.1. Security Analysis

In the presented solution, the achievement of precision in the access control is accomplished by employing smart contracts and applying attribute-based access control policies. Initially, RO exercises comprehensive authority over its extant resources. The pertinent resource details, encompassing addresses, attributes, and access permissions, undergo encryption via specified algorithms. Notably, the aforementioned process is devoid of any involvement from third-party entities in data acquisition and resource allocation. Furthermore, the incorporation of blockchain technology facilitates distributed access control. Blockchain serves as a communication medium among the three entities, which encompass the storage of all resource records and transactional information. The inherent features of blockchain technology ensure the simultaneous achievement of traceability and immutability in resource utilization.

Anonymity

In this scheme, the IV is ascertained through attributes rather than the user’s actual identity, thus providing the system with a level of anonymity. Each allocation of resource sharing involves the assignment of a set of attributes and their corresponding private keys to individual IVs. When instantiated VNFs seek access to resources, the system validates the user’s attribute set and private key. Access is granted only when all conditions are satisfied, thereby ensuring the preservation of VNFs’ genuine identity privacy while facilitating robust control over resource access.

Conversely, in conventional ABE schemes, the access policy is directly appended to the ciphertext in plaintext. This practice compromises the confidentiality of relevant technical details by making access policies susceptible to exposure. In this research, an enhanced Bloom filter is employed to obfuscate the correlation between attributes and access structures. Upon the initiation of resource sharing, the system computes the attribute set of the IV. If the attribute set aligns, the policy rho can be reconstructed; otherwise, an error message is generated. This approach effectively conceals the access policy associated with each shared resource.

On-repudiation attack:

In the aforementioned scheme, whenever there is a need for resource sharing allocation, a smart contract is invoked to verify permissions. Subsequently, a reliable and unchangeable access log is recorded on the blockchain. Any unauthorized access conducted by malicious IVs will be meticulously recorded on the blockchain, thereby rendering non-repudiation unattainable.

Man-in-the-middle attack:

A potential vulnerability arises in the context of man-in-the-middle attacks, wherein a malevolent entity illicitly interferes with the communication exchange among the RO, IP, and IV. In the proposed scheme, the algorithm integrates attribute permission authentication for the authorization requests initiated by the IV. This mechanism serves as an supplementary safeguard, even in scenarios where a malicious IV initiates an authorization request.

The safeguarding process entails the verification of attributes presented in a request by the smart contract to ensure their alignment with the access policy defined by the RO. Furthermore, the request necessitates the inclusion of a unique IV identifier, which is securely documented on the blockchain ledger before transmission. Through the verification procedures embedded in the smart contract, any tampered or falsified requests can be readily detected, thereby enhancing the security against potential man-in-the-middle attacks.

#### 7.3.2. Algorithm Performance Comparison

In order to assess the effectiveness of the proposed CP-ABE access control method, a comparative analysis of the efficiency of encryption and decryption processes was conducted. The traditional CP-ABE [42] and the improved Multiauthority CP-ABE [43] were used as benchmarks for comparison. The authors of [43] propose a methodology aimed at realizing nuanced access control within Internet of Things (IoT) healthcare systems. The aforementioned objective is accomplished by employing Elliptic Curve Cryptography (ECC) and CP-ABE. The employed strategy entails integrating multiple Attribute Authorities (AAs) to distribute user keys, effectively addressing the key escrow predicament typically associated with a single authority. In addition, the chosen methodology opts for ECC instead of bilinear pairing operations, leading to reduced computational and communication expenses. Furthermore, the introduction of User Assistant Entities (DUA) is pivotal, as they facilitate the outsourcing of specific components of the decryption process. This innovation effectively alleviates the decryption burden on users.

For convenience, the method proposed in this paper is abbreviated as BE, the traditional CP-ABE algorithm is referred to as TE, and the Multiauthority CP-ABE method is denoted as ME. We evaluate the efficiency of the encryption and decryption processes under various resource attribute quantities.

**Figure 10:**
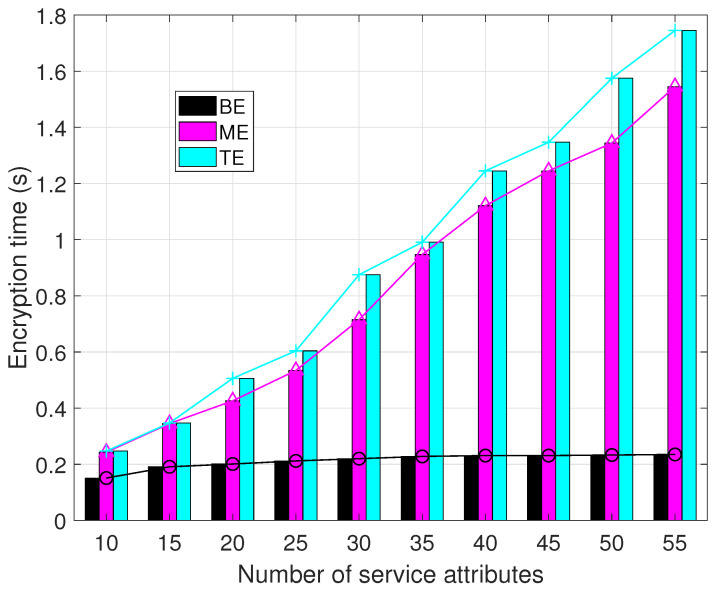
Encryption time comparison.

In Figure 10, it is apparent that the proposed solution demonstrates a gradual growth in encryption time with the augmentation of resource attributes. In contrast, the other two algorithms exhibit varying degrees of increased encryption times, with the TE method consuming the highest amount of time.

The occurrence of this phenomenon can be attributed to the inclusion of extensive precomputation procedures within the BE. The strategic implementation of precomputation enables the efficient generation of ciphertext upon obtaining resource information. As a result, the proliferation of attributes has a minimal impact on the efficiency of encryption for BE.

Conversely, ME leverages more efficient ECC algorithms in lieu of bilinear pairing operations. Consequently, this method exhibits superior encryption efficiency compared to TE.

**Figure 11:**
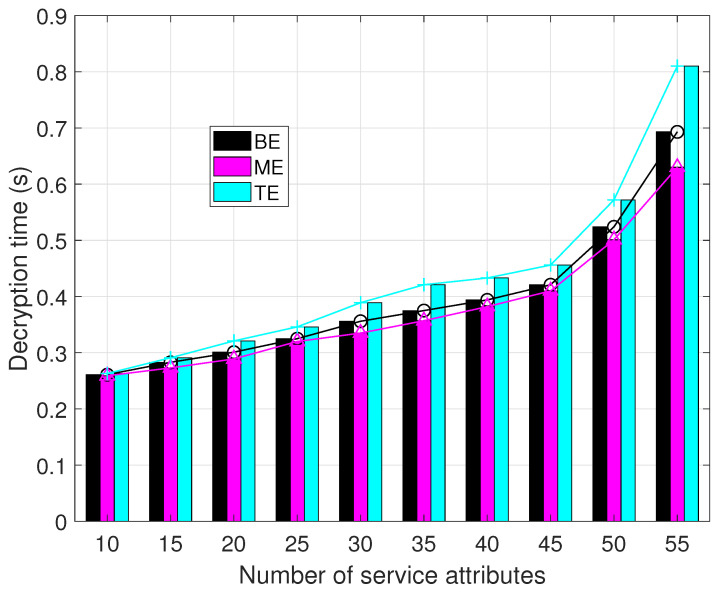
Decryption time comparison.

From Figure 11, it becomes evident that the decryption times for all three methods exhibit a substantial increase as the number of resource attributes increases. Among them, the ME method exhibits the shortest decryption time, followed by our proposed BE method, while the TE method experiences the longest decryption time.

This discrepancy is due to the utilization of a Bloom filter to conceal the resource access policies in this paper, which requires invoking the Bloom function to reconstruct IV’s access policies before decryption. This additional component increases the overall decryption time. Conversely, the remarkable efficiency of the ME method can be attributed to its utilization of DUA to facilitate partial decryption. The delegation of decryption responsibilities mitigates the decryption workload of the system, resulting in the highest level of decryption efficiency among the evaluated methods.

In summary, the method proposed in this paper enhances security within a blockchain-based framework while maintaining excellent encryption efficiency and moderate decryption efficiency.

## 8. Conclusions

This paper introduces a blockchain-based framework designed to facilitate efficient VNF resource sharing and implement secure access control. Our proposed approach aims to optimize the benefits for both infrastructure providers and VNF instances. To achieve optimal resource utilization, we present a resource sharing game model and a corresponding matching algorithm. Moreover, our innovative access control mechanism ensures secure key storage and enables fine-grained access control. Simulation results confirm the efficiency and superiority of our proposed solutions.

Nevertheless, an aspect that has not been addressed in this study pertains to the predictability of network traffic. The integration of sophisticated machine learning algorithms in the prediction of network traffic has the potential to offer resource owners a more comprehensive understanding, thereby facilitating the formulation of enhanced pricing strategies. Therefore, future work could prioritize the incorporation of machine learning techniques in the realm of business and traffic prediction, which offers a supplementary data support for the resource sharing game model. Furthermore, this paper exclusively employs a negative correlation as a parameter in the utility function to model relationships among VNFs. To enhance utility functions and improve resource sharing strategies, future work will explore the integration of traffic sequencing among multiple VNFs and investigate the potential correlation among various business entities.

## Figures and Tables

**Table 1:** Variable notations of Resource Sharing Model.

Symbol	Description
$r_{i}$	The demand for resource *i*
$\mu_{i}$	The unit cost of resource *i* for IP
$C_{P}^{i}$, $C_{P}$	The cost of resource *i* and the total cost of VNF
$\sigma_{j}$	The degree of relevance between IV and $RO_{j}$
$\upsilon_{i}$	The utility level of resource *i*
$r_{i}^{0},r_{i}^{j}$	The variable whether resource *j* is deployed on a shared node $RO_{j}$ or as an exclusive resource
$U_{i}^{j}$	The utility function for deploying resource *i* to $RO_{j}$
$F_{i}^{j}$	The available quantity of resource *i* within $RO_{j}$ during the available time period
$\rho_{i}^{j}$	The unit price for resource *i* in $RO_{j}$
$\kappa_{i}$	The unit retail price for resource *i* of IP
$P_{I}$	The sum resulting profit for IP
$\lambda_{i}^{j}$	The cost level of $RO_{j}$ for resource *i*
$C_{i}^{j}$	The Cost function for deploying resource *i* to $RO_{j}$
$P_{R}^{j}$	The profit of the $RO_{j}$
$RS_{i}^{j}$	Resource *i* is shared with $RO_{j}$
$NRS_{i}$	Resource *i* is allocated by an exclusive resource

**Table 2:** Major simulation parameters.

Parameter	Value
Number of ROs	Random from 1 to 50
Resource requirement vector lengths	Random from 1 to 10
$r_{i}$	Random from 1 to 50
$\sigma_{j}$	Uniform distribution, ranging from 0.1 to 0.9
$\lambda_{i}^{j}$	Uniform distribution, ranging from 0.1 to 0.9
$\mu_{i}$	Random from 100 to 300
$\kappa_{i}$	25% to 35% higher than $\mu_{i}$ randomly

## Data Availability

Data are contained within the article.
